# Efficacy of Thiocolchicoside for Musculoskeletal Pain Management: A Systematic Review and Meta-Analysis of Randomized Controlled Trials

**DOI:** 10.3390/jcm13206133

**Published:** 2024-10-15

**Authors:** Alessandro Bianconi, Matteo Fiore, Annalisa Rosso, Cecilia Acuti Martellucci, Giovanna Letizia Calò, Giovanni Cioni, Gianmarco Imperiali, Vittorio Orazi, Marco Tiseo, Anastasia Troia, Enrico Zauli

**Affiliations:** 1 School of Public Health, Department of Medical and Surgical Sciences, University of Bologna, Via San Giacomo 12, 40138 Bologna, Italy; alessandro.bianconi4@studio.unibo.it (A.B.); matteo.fiore7@studio.unibo.it (M.F.); 2 Department of Environmental and Prevention Sciences, University of Ferrara, 44121 Ferrara, Italy; annalisa.rosso@unife.it (A.R.); giovannaletizia.calo@edu.unife.it (G.L.C.); giovanni01.cioni@edu.unife.it (G.C.); gianmarco.imperiali@edu.unife.it (G.I.); vittorio.orazi@edu.unife.it (V.O.); marco.tiseo@edu.unife.it (M.T.); anastasia.troia@edu.unife.it (A.T.); 3 Department of Translational Medicine, University of Ferrara, 44121 Ferrara, Italy; enricozauli8@gmail.com

**Keywords:** thiocolchicoside, musculoskeletal pain, relaxant, pain management

## Abstract

**Background**: Despite the fact that thiocolchicoside has been widely used in the treatment of musculoskeletal pain, its efficacy has never been systematically evaluated. We carried out a systematic review and meta-analysis of randomized clinical trials (RCTs) to appraise the existing evidence on the efficacy of thiocolchicoside for musculoskeletal pain management. **Methods**: The literature search was performed on multiple databases, extracting reports of RCTs evaluating the efficacy of thiocolchicoside compared to placebo or no exposure. The reduction in pain intensity was evaluated through between-groups mean differences (MDs) in Visual Analogue Scale (VAS) scores after the intervention. The pooled effect estimates were compared to a minimally important difference (MID) of 1 point on a scale from 0 to 10. **Results**: We retrieved eight RCTs, including a total of 1397 patients. All the included studies were determined as being at high risk of bias. Seven trials evaluated patients with acute low back pain. After 2–3 days of treatment, the pooled MD in VAS score was −0.49 (95%CI = −0.90; −0.09) compared to controls. After 5–7 days of treatment, the summary MD was −0.82 (95%CI = −1.46; −0.18). **Conclusions**: Although thiocolchicoside was found to significantly reduce patient-reported low back pain, the clinical impact was very small, as the pooled effect estimates were below the MID, and the overall certainty of evidence was very low. In light of the safety concerns raised by the European Medicine Agency, an in-depth analysis on the risk–benefit balance of thiocolchicoside is required.

## 1. Introduction

Thiocolchicoside is a pharmacological compound used in clinical practice as a muscle relaxant in the treatment of painful musculoskeletal disorders, such as acute lower back pain, neck pain, and other conditions that cause muscle stiffness and discomfort [1]. Because of safety concerns [1,2,3,4], in 2013, the European Medicine Agency (EMA) recommended restricting the use of this drug [2]. In particular, potential genotoxic effects, along with known epileptogenic and hepatoxic activity, have been reported [1,2,3,4], leading to the decision to limit the use of injective or oral drugs containing thiocolchicoside to adjuvant therapy for the short-term relief of painful muscle contractures [2]. However, thiocolchicoside-containing drugs are still widely used in clinical practice [5].

Some trials reported thiocolchicoside effects in relieving pain in musculoskeletal conditions [6,7,8,9], but no systematic reviews quantifying the overall efficacy are available. Moreover, it is unclear whether the effects on pain management reported in the literature are clinically meaningful, for example, when compared to a minimally important difference (MID), which represents the smallest change in a treatment outcome that patients perceive as beneficial [10,11]. Without a clear understanding of the efficacy of thiocolchicoside in the management of musculoskeletal pain, it is difficult to assess whether the use of this treatment remains justified, given the safety concerns raised by the drug regulatory agencies. Thus, we carried out a meta-analysis to summarize and appraise the existing evidence derived from randomized clinical trials (RCTs).

## 2. Materials and Methods

The review protocol was registered on PROSPERO (CRD42024568209), and the results have been reported according to PRISMA guidelines [12]. The systematic literature search was performed on PubMed, Scopus, MedRXiv, ClinicalTrials.gov, and the WHO International Clinical Trials Registry Platform databases. We searched for articles and conference proceedings reporting the results of RCTs evaluating the efficacy of thiocolchicoside versus placebo or no exposure in patients with musculoskeletal pain conditions. When the intervention was represented by thiocolchicoside + another drug, the study was considered eligible if the comparator was placebo + the other drug, or the other drug alone. All outcomes of efficacy were considered eligible. No restrictions were placed on the geographic origins or languages of the articles. The references of the included studies were also searched for additional studies. For the search, we used keywords regarding thiocolchicoside, combining, using Boolean logic, ‘AND’ and ‘OR’ (last search update: 4 July 2024). The detailed search strategy is provided in the Supplementary Material (Table S1).

After the exclusion of duplicates, the eligibility of each retrieved study was assessed by two authors (AB and MF) independently and blindly by screening titles and abstracts using SysRev [13]. Any discrepancy between the authors was solved via discussion with a third author (CAM), and the complete list of excluded articles with reasons for exclusion is reported in Supplementary Table S2. The methodological quality of each article was evaluated using the Risk of Bias 2 tool for RCTs [14]. The Cochrane RoB 2 tool was chosen for its robust framework in assessing bias in randomized trials [14,15], while acknowledging that several other risk-of-bias evaluation tools exist, each with limitations due to variations in the specific domains that they address, the fixed nature of their criteria, and differing approaches to bias assessment [16,17]. The GRADE approach was used to determine the certainty of evidence for each extracted outcome [11].

The main outcome that we examined was the reduction in pain intensity, evaluated through the use of patient-reported Visual Analogue Scale (VAS) scores after the intervention. Other measures of pain intensity were also extracted, including physician-reported VAS, pain pressure threshold score, pain during movement score, and the presence of muscle spasms. Moreover, we also extracted measures of functional impairments, including hand-to-floor distance, Schober’s test score [18], patient-reported global evaluation scores, and scores for reduction in active and passive segmental mobility.

For each study, if VAS scores were reported on a different scale (e.g., 0–10, or 0–100), they were converted to a 0–10 scale and we computed the mean difference (MD) between the intervention and control group using the mean VAS scores and standard deviations (SDs) at comparable time points. When a paper did not specifically describe whether the VAS scores were patient- or physician-reported, we assumed that they were patient-reported. In the case of missing numerical data or SDs, we tried to contact the authors. If no response was obtained, the outcomes that were not reported as numerical data but only in graphical form were estimated through the visual inspection of the graphs [19], while SDs were imputed using the pooled SDs of the other studies included [20], following the methods described by Furukawa et al. [15,21]. Data from single studies were meta-analyzed using the random-effects inverse variance approach to account for between-study variance, computing the summary of VAS score mean differences, with 95% confidence intervals (CIs), and evaluating the between-study heterogeneity through the use of I^2^ statistics. Meta-analyses were performed when three or more studies of similar design and follow-up assessing the same outcome were available and were presented stratifying by administration routes. Sensitivity analyses were performed excluding studies with imputed data or with topical and/or oral preparations, to account for heterogeneity in the data quality and administration routes of the included studies, as suggested by the Cochrane Handbook for Systematic Reviews of Interventions [15]. When specific outcomes could not be meta-analyzed due to insufficient comparable data (e.g., outcome evaluated in only one study), a narrative synthesis was provided by summarizing the effect estimates though the tabulation of the available data [15]. All analyses were performed using Review Manager 5.4 [22]. The pooled effect estimates were compared to an MID to assess whether a clinically important effect was present [10]. In particular, the MID threshold was used to evaluate the consistency of the evidence, rather than relying on the null threshold [10,23]. As described in a recent systematic review, different MID thresholds for the VAS pain scores are reported in the literature, differing by the type of setting and condition assessed, and ranging between 0.8 and 4 points on a scale from 0 to 10 [24]. We decided to set a conservative MID threshold of 1 point, as this is frequently used for VAS values [23] and is also the threshold most often reported in validation studies [24].

## 3. Results

**Figure 1:**
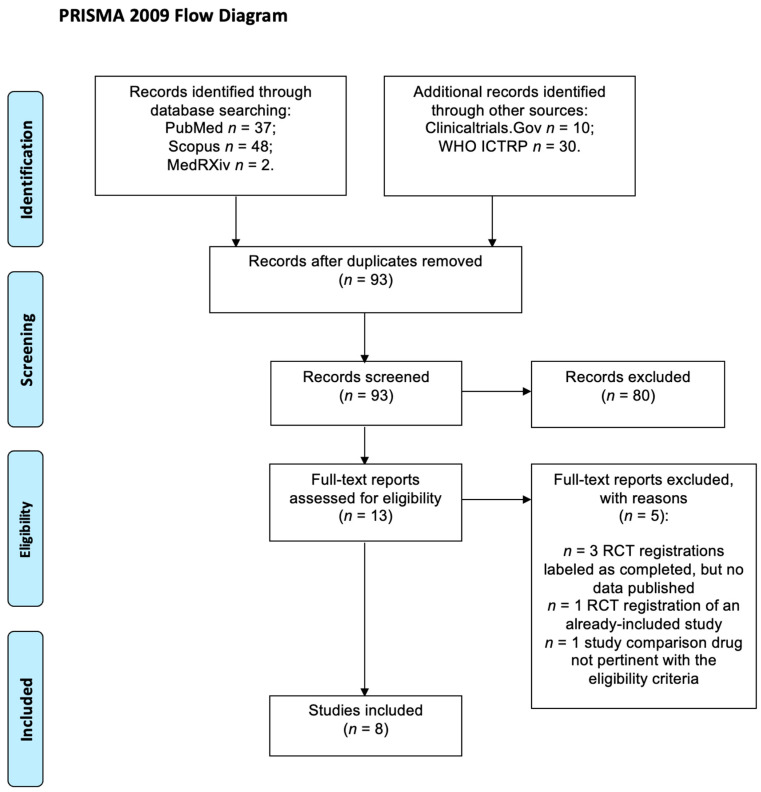
PRISMA flowchart describing the selection process for the included studies.

The initial search identified 127 reports; 34 were removed because they were duplicates, and 80 were excluded during the title/abstract screening stage. The remaining 13 articles were assessed for eligibility by reading the full text, and a total of eight RCTs [6,7,8,9,19,20,25,26] met the criteria for final inclusion (Figure 1). The summary of the characteristics of the included studies is reported in Table 1: only four were published in the last 20 years; the vast majority (*n* = 7) evaluated the efficacy of thiocolchicoside in patients with acute low back pain. The working definition of low back pain varied across the included studies, with heterogeneous entry criteria regarding the duration and the intensity of pain (Table S3). The outcomes were mainly measures of pain intensity, including patient- or physician-reported VAS scores at various time points, pain pressure threshold score, pain during movement score, and the presence of muscle spasms. The only RCT that evaluated the efficacy of thiocolchicoside in a sample with a different condition—patients with osteoarthritis—reported the efficacy of the drug in terms of a reduction in active and passive segmental mobility. We also extracted the available safety data (Table S4): only one serious adverse event was reported in the control group (hospitalization due to chest pain, with no complications), all other events were reported as mild and balanced between the intervention and control group.

**Figure 2:**
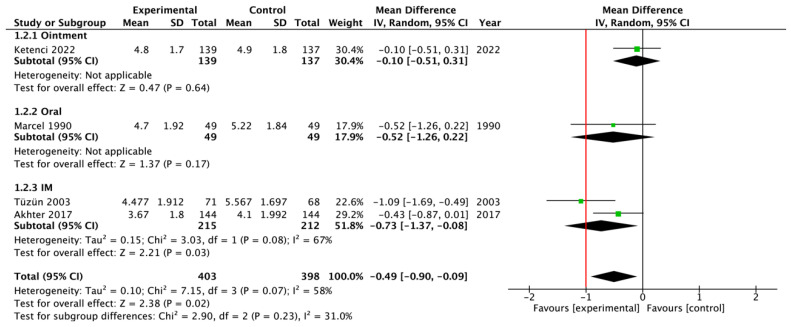
Forest plot regarding the meta-analysis of mean differences in efficacy of thiocolchicoside on Visual Analogue Scale scores at 2 or 3 days from the start of the treatment, in patients with acute low back pain [6,8,9,25]. The red vertical line represents the minimal important difference (MID) of 1 point. SD, standard deviation; CI, confidence interval.

**Figure 3:**
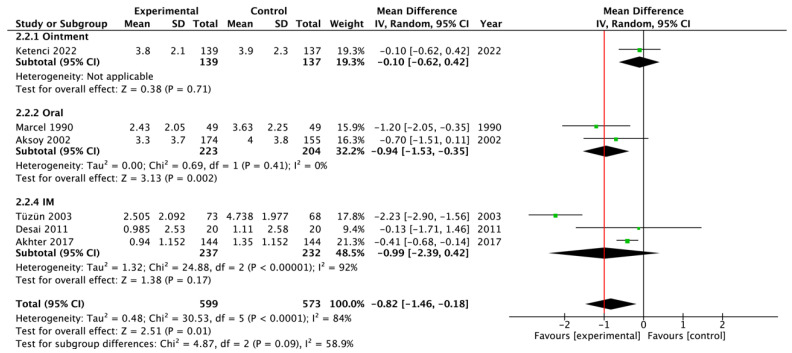
Forest plot regarding the meta-analysis of mean differences in the efficacy of thiocolchicoside in Visual Analogue Scale scores at 5 or 7 days from the start of the treatment in patients with acute low back pain [6,8,9,19,20,25]. The red vertical line represents the minimal important difference (MID) of 1 point. SD, standard deviation; CI, confidence interval.

Seven RCTs evaluated patients with acute low back pain and compared thiocolchicoside to no treatment or placebo, measuring the mean VAS scores before and after the intervention. Four studies (*n* = 801 patients) provided data at an intermediate time point (2/3 days from the start of the treatment), and six studies (*n* = 1172 patients) provided data at the end of the therapy (5/7 days from the start of the treatment). The pooled estimates were MD = −0.49 (95% CI = −0.90, −0.09; *p* < 0.05) after 2/3 days (Figure 2) and MD = −0.82 (95% CI = −1.46, −0.18; *p* < 0.05) at the end of the treatment (Figure 3). The route of administration of thiocolchicoside differed among the studies included in the meta-analyses: intramuscular injections (*n* = 3 RCTs), oral tablets or capsules (*n* = 2), or topical ointment (*n* = 1). In the sensitivity analyses excluding the studies in which the drug was administered topically or orally, or the RCTs with some imputed data, the results did not substantially differ (Figures S1–S7). In the single study in which thiocolchicoside was administered topically, however, the drug showed no benefit over the placebo.

All the other outcomes were not evaluated in a sufficient number of studies (less than 3) to perform a meaningful meta-analysis; therefore, a narrative synthesis of the individual results is reported in Table 2.

**Figure 4:**
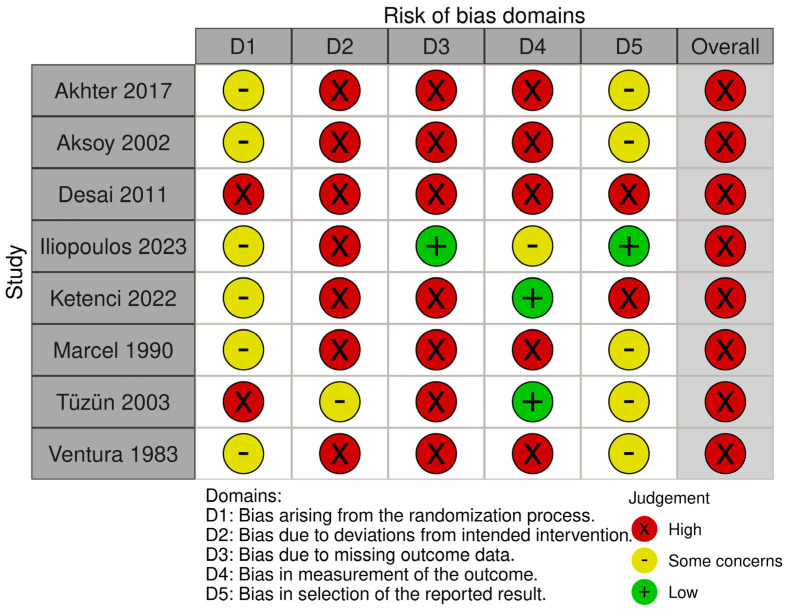
Risk of bias assessment [6,7,8,9,19,20,25,26].

All the included RCTs were judged as having a “high” overall risk of bias (Figure 4). The most common sources of bias concerns were inadequate or insufficiently described allocation concealment procedures (100% of the RCTs), inadequate or insufficiently described methods to handle deviations from the intended interventions (100%), and inadequate or insufficient information on missing outcome data (87.5%). Following the GRADE approach, the overall certainty of evidence for all the evaluated outcomes of the efficacy of thiocolchicoside for musculoskeletal pain was judged to be very low (Table 2) due to the high risk of bias, inconsistency (substantial heterogeneity and variability in single-study point estimates falling above or below the MID threshold), and imprecision (wide 95% CI, in the case of MDs of VAS scores, CI always included or lower than the MID threshold). The risk of publication bias was unclear: as the systematic review included a small number of studies, publication bias could not be assessed using funnel plots, nor formally tested through Egger’s test [15]. However, a potential file-drawer effect was found due to the presence of registered and completed RCTs, whose results were not published [27,28,29].

## 4. Discussion

Despite thiocolchicoside having been widely used for musculoskeletal pain management for decades [2,5], no meta-analysis has been published on its efficacy; only eight RCTs could be found—seven of which focused on low back pain only—and all of the available trials were rated as having a high risk of bias due to the inadequate or insufficient reporting of allocation concealment measures, methods to handle deviations from the intended interventions, and missing outcome data [14]. In addition, and most importantly, although the oral or intramuscular administration of thiocolchicoside was able to significantly reduce patient-reported low back pain in most analyses, the clinical impact was very small, as the pooled estimate of effect was always below the minimally important difference of a 1-point reduction on a 0–10 VAS.

These findings highlighted the need for an in-depth analysis of the risk–benefit balance in thiocolchicoside, as potentially harmful side effects emerged over the years from pharmacovigilance investigations [2,4]. In addition to potential epileptogenic and hepatoxic activity [1,2,30,31], an EMA investigation issued some precautionary limitations on the drug use during pregnancy because of a risk of teratogenicity and embryonal and fetal toxicity and recommended an overall duration of the treatment of a maximum of 5 or 7 days for IM or oral administration, respectively [2]. Although a recent study reported that the risk minimization measures promulgated by EMA seemed to have had an impact on off-label prescription practices among European physicians, the general approved use of thiocolchicoside appears to have remained high [5]; thiocolchicoside-containing drugs were ranked 18th in the most sold Class C medicines with prescription in Italy in 2022 [32].

These results are in line with the current overall low efficacy of treatments used for low back pain [33,34]. Still, several pharmacological alternatives with higher certainty of evidence exist [33,35]. For example, two Cochrane reviews reported the use of both topical and systemic nonsteroidal anti-inflammatory drugs as being effective in providing musculoskeletal pain relief [33,35]. Given these premises, a group of members of the EMA committee that reviewed recommendations of thiocolchicoside signed a divergent statement highlighting that until further safety reports emerged, the use of thiocolchicoside did not appear justified, since alternative options without genotoxic effects were available [2]. Considering the whole scenario, an independent, comprehensive risk–benefit analysis is needed.

To our knowledge, this study provides the first systematic assessment of the efficacy of thiocolchicoside, adding quantitative parameters on the extension, reliability, and clinical impact of the existing evidence on the topic. Based on the results of this review, the efficacy of thiocolchicoside appeared to be clinically marginal, with a very low level of certainty of evidence. These results highlight the need for further research and may enable healthcare professionals to make better-informed decisions regarding the use of this drug, particularly in light of its potential safety risks. This study also has some limitations that must be considered in interpreting the results. First, the inclusion criteria selected only RCTs, leaving out other potential non-randomized studies. However, it is well known that the lack of randomization in group allocation can lead to strongly biased results, especially in the evaluation of a drug analgesic effect [15]. Second, the vast majority of the studies were based on participants with low back pain. Although a generalization of the interpretation of the results can be meaningful for all acute idiopathic algic conditions of the spine, patients with low back pain may differ from those with other musculoskeletal conditions, and, thus, the effects of thiocolchicoside in other populations may differ, too. Third, a substantial heterogeneity was found in most meta-analyses, which, however, might reflect the differences across studies in administration routes, treatment durations, data quality, and participant inclusion criteria. Finally, all included studies were characterized by poor methodological and reporting quality, with a high risk of selection bias, unmasking and selective reporting that may have presumably caused a shift of the effect estimates in favour of the intervention, with an overall low certainty of effect.

## 5. Conclusions

In conclusion, the oral or intramuscular administration of thiocolchicoside resulted in a statistically significant reduction in patient-reported low back pain, but the clinical significance was marginal, as the pooled effect estimates were always below the minimally important difference of a 1-point reduction on a 0–10 VAS, which is unlikely to lead to meaningful clinical benefits for patients. The overall literature was limited, no meta-analyses could be performed on other outcomes or algic conditions, and all the included studies were at high risk of bias, with a potential shift in the pooled estimates to a favourable effect size. Further high-quality research, eventually evaluating alternative dosing regimens or specific combinations with other therapies, may clarify the role of thiocolchicoside in the management of musculoskeletal pain. As potentially harmful side effects have emerged over the years from pharmacovigilance investigations, an in-depth risk–benefit analysis of thiocolchicoside is needed to assess whether its widespread use over alternative drugs remains justified.

## Figures and Tables

**Table 1:** Characteristics of the included studies.

First Author	Year	Country	Funding	Study Population	Sample	Mean Age	% Female Participants	Intervention	Comparison	Outcomes and Effect Sizes
Akhter [6]	2017	Pakistan	Not reported	Adults (>18 y) with acute LBP with muscle spasms	288 ITT	Not reported	53	Thiocolchicoside (4 mg) + Diclofenac (75 mg) IM injection, twice daily	Diclofenac (75 mg) IM injection	Day 3 VAS: MD = −0.43 [−0.87, 0.01]
										Day 7 VAS: MD = −0.41 [−0.68, −0.14]
										Day 3 HTF distance: MD = −1.69 [−2.25, −1.13]
										Day 7 HTF distance: MD = −1.58 [−1.95, −1.21]
Aksoy [19]	2002	Turkey	Not reported	Adults (18–65 y) with acute or sub-acute LBP	372 ITT, 329 PP	40 ± 11 y	64	Thiocolchicoside capsules (8 mg), twice daily + NSAID	Standard treatment (NSAID or BDZ or corticosteroid)	Day 7 VAS: MD = −0.70 [−1.51, 0.11] *
										Day 31 VAS: MD = −0.50 [−1.28, 0.28] *
										Day 7 RMDQ scores: MD = −4.50 ^A^ *
										Day 31 RMDQ scores: MD = −5.00 ^A^ *
Desai [20]	2011	India	Not reported	Adults (18–55 y) with acute LBP	40 ITT, 40 PP	M: 39 y; F: 38 y	63	Thiocolchicoside (4 mg) + Aceclofenac (100 mg) IM injection, twice daily	Aceclofenac IM injection	Day 7 mean VAS: MD = −0.13 [−1.71, 1.46] **
										Day 7 mean pain during movement score: MD = −0.1 ^B^
										Day 7 mean movement restriction score: MD = −0.35 ^B^
Iliopoulos [7]	2023	Greece	Win Medica S.A.	Adults (>18 y) with acute LBP	134 ITT, 123 PP	52 ± 11 y	66	Thiocolchicoside (4 mg) + Diclofenac (75 mg) IM injection	Diclofenac (75 mg) IM injection	3 h VAS: MD = −1.30 [−1.87, −0.72]
										1 h VAS: MD = −0.36 [−0.98, 0.26]
										1 h, > 30% reduction VAS: RR = 1.50 [0.86, 2.65]
										3 h, > 30% reduction VAS: RR = 1.60 [1.03, 2.52]
										1 h HTF distance: MD = −0.92 [−6.79, 4.95]
										3 h HTF distance: MD = −4.55 [−9.66, 0.56]
Ketenci [25]	2022	Turkey	Multiple sponsors ^C^	Adults (18–64 y) with acute LBP with muscle spasms	292 ITT, 276 PP	39 ± 11 y	64	Thiocolchicoside oinment (0.25%)	Placebo	Day 3 PPT: MD = 0.10 [−0.29, 0.49]
										Day 7 PPT: MD = −0.20 [−0.65, 0.25]
										Day 3 VAS (patient-reported): MD = −0.10 [−0.51, 0.31]
										Day 7 VAS (patient-reported): MD = −0.10 [−0.62, 0.42]
										Day 3 VAS (physician-reported): MD = −0.10 [−0.49, 0.29]
										Day 7 VAS (physician-reported): MD = −0.10 [−0.60, 0.40]
										Use of paracetamol as rescue drug: RR = 0.77 [0.48, 1.23]
Marcel [8]	1990	France	Not reported	Patients (range not reported) with acute LBP	98 ITT, 94 PP	38 ± 10 y	38	Thiocolchicoside tablets (8 mg), twice daily	Placebo	Day 2 VAS: MD = −0.52 [−1.26, 0.22]
										Day 5 VAS: MD = −1.20 [−2.05, −0.35]
										Day 2 HTF distance: MD = −4.10 [−10.51, 2.31]
										Day 5 HTF distance: MD = −8.80 [−15.92, −1.68]
										Day 2 Schober Index: MD = −0.20 [−0.64, 0.24]
										Day 5 Schober Index: MD = −0.50 [−1.02, 0.02]
										Use paracetamol as rescue drug: MD = −3.70 [−7.07, −0.33]
										Patients with very good/good global evolution score: RR = 2.13 [1.36, 3.31]
Tüzün [9]	2003	Turkey	Not clearly reported ^D^	Adults (18–65 y) with acute LBP	143 ITT, 137 PP	41 ± 11 y	54	Thiocolchicoside IM injection (4 mg), twice daily	Placebo	Day 3 VAS: MD = −1.09 [−1.69, −0.49]
										Day 5 VAS: MD = −2.23 [−2.90, −1.56]
										Patients with no spasms at day 5: RR = 1.92 [1.19, 3.09]
										Use of paracetamol as rescue drug: MD = −2.5 ^B^
										Patients with very good/good global evolution score: RR = 2.89 [1.89, 4.42]
Ventura [26]	1983	Italy	Not clearly reported ^E^	Patients with coxarthrosis, gonarthrosis, scapulohumeral periarthritis	30 ITT	Not reported	Not reported	Thiocolchicoside capsules (8 mg), twice daily	Placebo	Day 5 reduction ASM: MD = −10.24 [−18.90, −1.58]
										Day 10 reduction in ASM: MD = −18.92 [−27.20, −10.64]
										Day 5 reduction in PSM: MD = −10.74 [−19.53, −1.95]
										Day 10 reduction in PSM: MD = −17.86 [−25.70, −10.02]

* LBP, low back pain; ITT, intention-to-treat; PP, per protocol; IM, intramuscular; PPT, pressure point threshold score; MD, mean difference; VAS, visual analogue scale for pain; HTF, mean hand-to-floor; RR, rate ratio; NSAID, nonsteroidal anti-inflammatory drug; BDZ, benzodiazepine; RMDQ, Roland–Morris disability questionnaire; M, male; F, female; ASM, active segmental mobility. PSM, passive segmental mobility. * Data estimated through the visual inspection of the graphs. ** Standard deviations imputed using the pooled SDs of the other included studies. ^A^ Unspecified measure of variability. ^B^ Standard deviation not reported. ^C^ Turkish Medicines and Medical Devices Agency, Abdi İbrahim Pharmaceuticals, Avixa Pharma, Bilim Pharmaceuticals, Nobel Pharmaceuticals, Pharma Dor Pharmaceuticals, Sanovel Pharmaceuticals, Santa Farma, World Medicine Pharmaceutical were responsible for the preparation and the supply of study products. ^D^ Drug supplies were prepared by Sanofi-Synthelabo. ^E^ Drug supplies were prepared by the company Ditta Inverni, Beffa, Milan.

**Table 2:** Summary of the findings.

Certainty Assessment							№ of Patients		Effect		Certainty	Importance
№ of Studies	Study Design	Risk of Bias	Inconsistency	Indirectness	Imprecision	Other Considerations	Thiocolchicoside	Placebo/No Treatment	Relative (95% CI)	Absolute (95% CI)		
**Low Back Pain—Intensity (follow-up ranging from 2 days to 3 days, assessed with Visual Analogue Scale from 0 to 10)**												
4	Randomized trials	Very serious ^a^	Serious ^b^	Not serious	Serious ^c^	None	403	398	-	MD **0.49 lower** (0.9 lower to 0.09 lower)	⨁◯◯◯ Very low	
**Low Back Pain—Intensity (follow-up ranging from 5 days to 7 days, assessed with Visual Analogue Scale from 0 to 10)**												
6	Randomized trials	Very serious ^a^	Serious ^b^	Not serious	Serious ^c^	None	599	573	-	MD **0.82 lower** (1.46 lower to 0.18 lower)	⨁◯◯◯ Very low	
**Low Back Pain—Intensity (assessed with other measures, including Visual Analogue Scale for Pain at 1 h and 3 h; Visual Analogue Scale scores at day 31; pain pressure threshold; the use of paracetamol as a rescue drug, physician-reported Visual Analogue Scale for pain scores at Day 3 and Day 7; the presence of muscle spasms)**												
5	Randomized trials	Very serious ^a^	Not serious	Not serious	Serious ^d^	None	Two studies found favourable effects of thiocolchicoside on other pain intensity outcomes (the presence of muscle spasms and the mean use of paracetamol as a rescue drug), two studies found null effects of thiocolchicoside on other pain intensity outcomes (Visual Analogue Scale scores at day 31, pain pressure threshold, the use of paracetamol as a rescue drug, and physician-reported Visual Analogue Scale for pain scores at Day 3 and Day 7). One study found null effects from a single administration of thiocolchicoside on Visual Analogue Scale scores after 1 h, and statistically significant, but very small, effects on Visual Analogue Scale scores after 3 h (upper limits of the 95% CIs were above the minimally important difference threshold).				⨁◯◯◯ Very low	
**Low Back Pain—Functional impairment (assessed with hand-to-floor distance; Schober’s test score; patient-reported global evaluation scores)**												
4	Randomized trials	Very serious ^a^	Serious ^e^	Not serious	Serious ^d^	None	Two studies found favourable effects on functional impairment outcomes (ratio of patients with very good/good global evolution score, and hand-to-floor distance at days 3 and 7). One study found mixed favourable effects on functional impairment outcomes (hand-to-floor distance at day 5) and null effects on functional impairment outcomes (hand-to-floor distance at day 2, Schober Index at days 2 and 5, and ratio of patients with very good/good global evolution score). One study found null effects on functional impairment outcomes (hand-to-floor distance at 1 and 3 h after a single administration).				⨁◯◯◯ Very low	
**Osteoarthritis—Functional impairment (assessed with a reduction in active segmental mobility score; reduction in passive mobility score)**												
1	Randomized trials	Very serious ^a^	Not serious	Serious ^f^	Not serious	None	One study found favourable effects on functional impairment outcomes (reduction in active segmental mobility at days 5 and 10, and reduction in passive segmental mobility at days 5 and 10).				⨁◯◯◯ Very low	

CI, confidence interval; MD, mean difference. Explanations: ^a^ Risk of bias evaluated as “very serious” due to the fact that all the included studies were assessed as at high risk of bias using the Cochrane RoB 2 tool. ^b^ Rated down for inconsistency due to the substantial heterogeneity and the variability in single-study point estimates falling above or below the minimally important difference (MID) threshold. ^c^ Rated down for imprecision due to wide 95% confidence intervals (CIs) always including, or being lower than, the MID threshold. ^d^ Rated down for imprecision due to wide 95% confidence intervals that often included the null or the MID threshold. ^e^ Rated down for inconsistency due to the variability in single-study point estimates falling above or below the MID threshold. ^f^ Rated down for indirectness because the population only included three different osteoarthritis conditions (hip, knee, and shoulder) and the treatment duration were too low for assessing the potential efficacy in this condition.

## Data Availability

All data are available from the studies included in the meta-analysis.

## Supplementary Materials

The following supporting information can be downloaded at: https://www.mdpi.com/article/10.3390/jcm13206133/s1, Figure S1: Sensitivity analysis excluding results derived by topical administration from the thiocolchicoside efficacy on VAS scores at 2–3 days time-point meta-analysis; Figure S2: Sensitivity analysis excluding results derived by topical administration from the thiocolchicoside efficacy on VAS scores at 5–7 days time-point meta-analysis; Figure S3: Sensitivity analysis excluding results derived by oral administration from the thiocolchicoside efficacy on VAS scores at 2–3 days time-point meta-analysis; Figure S4: Sensitivity analysis excluding results derived by oral administration from the thiocolchicoside efficacy on VAS scores at 5–7 days time-point meta-analysis; Figure S5: Sensitivity analysis excluding imputed results from Aksoy 2002 from the thiocolchicoside efficacy on VAS scores at 5–7 days time-point meta-analysis; Figure S6: Sensitivity analysis excluding imputed results from Desai 2011 from the thiocolchicoside efficacy on VAS scores at 5–7 days time-point meta-analysis; Figure S7: Sensitivity analysis excluding imputed results from Aksoy 2002 and Desai 2011 from the thiocolchicoside efficacy on VAS scores at 5–7 days time-point meta-analysis; Table S1: Detailed search strategy for each database; Table S2: List of reports excluded after the full-text screening process and reasons of exclusion; Table S3: Inclusion characteristics of patients with low back pain within the included studies; Table S4: Frequency of adverse events in the included studies.
