# Supplementary material for: Efficacy of Thiocolchicoside for Musculoskeletal Pain Management: A Systematic Review and Meta-Analysis of Randomized Controlled Trials
Source: J Clin Med. 2024 Oct 15;13(20):6133. doi: 10.3390/jcm13206133 (PMC11508309; doi:10.3390/jcm13206133)

Supplementary Material

Table S1. Detailed search strategy for each database.

Database	Search string
PubMed	"thiocolchicoside"[tiab] AND (random* OR trial)
Scopus	TITLE-ABS ("thiocolchicoside") AND (TITLE-ABS (random*) OR TITLE-ABS (trial))
MeRXiv	thiocolchicoside
ClinicalTrials.gov	thiocolchicoside
WHO ICTRP	thiocolchicoside

Table S2. List of reports excluded after the full-text screening process and reasons of exclusion.

Reference	Trial ID	Reason for exclusion
Thiocolchicoside Injection and Capsule in Treatment of Acute Low Back Pain	NCT00917436	Completed, but no data published
An Investigator initiated study comparing individual use of Etorocoxib (to reduce pain and swelling) and Thiocolchicoside (a muscle relaxant) against combined use of Etoroxocib and Thiocolchicoside in patients with pain in different regions of the spine along with muscle rigidity and spams.	CTRI/2023/08/056416	Completed, but no data published
A multiple centre study to check the efficacy and safety of Topical formulation of Diclofenac plus Thiocolchicoside in comparison with Diclofenac gel in acute low back pain.	CTRI/2011/11/002116	Completed, but no data published
This Phase III study will compare the efficacy and tolerability of a combination IM product of Thiocolchicoside and Diclofenac vs. Diclofenac (Voltaren®) IM in patients with acute low back pain	EUCTR2017-004530-29-GR	RCT registration of an already included study (Iliopoulos 2023)
Lanfranchi R, Volpi A. Risultati della sperimentazione clinica per via intrarticolare del tiocolchicoside nella periartrite scapolo-omerale [Results of the clinical trial of intra-articular thiocolchicoside in scapulo-humeral periarthritis]. Minerva Med. 1968;59(87):4678-4681.	n/a	Comparison drug not pertinent with the eligibility criteria (corticosteroid)

Table S3. Inclusion characteristics of patients with low back pain within the included studies.

Authors	Definitions of LBP and patients' inclusion criteria
Akhter 2017 [6]	LBP is defined as pain with muscle spasms interfering daily activities. Patients with a VAS pain scores equal to or greater than 50 mm were recruited in the study.
Aksoy 2002 [19]	Patients with an acute episode of recurrent LBP and with a VAS pain score equal to or greater than 50 mm were recruited in the study.
Desai 2011 [20]	Not reported.
Iliopoulos 2023 [7]	Patients with acute, moderate to severe LBP, defined as VAS pain score equal to or greater than 40 mm were recruited in the study.
Katenci 2022 [25]	Patients with LBP lasting more than six weeks and/or detection of an acute muscle spasm on physical examination were recruited in the study.
Marcel 1990 [8]	Patients with uncomplicated LBP with a VAS pain score equal to or greater than 50 mm, with functional impairment in daily activities, and a fingertip-to-floor distance of 30 cm or more.
Tüzün 2003 [9]	Patients with LBP with a VAS pain scores equal to or greater than 50 mmm and with severe or moderate lumbar muscle spam lasting less than 72h.

LBP = Low Back Pain; VAS = Visual Analogue Scale.

Table S4. Frequency of adverse events in the included studies

Study	Experimental AEs n (%)	Control AEs n (%)	Description of safety data *
Akhter 2017 [6]	6 (4%)	-	Complaint of sedation none. Complaint nausea vomiting abdominal discomfort few patients in both groups. Diarrhea reported by 6 patients in the intervention group [17].
Aksoy 2002 [19]	11 (6%)	6 (4%)	No patient reported drowsiness. There were no withdrawals because of adverse events, and no serious adverse events were reported. A total of 10 patients in the “thiocolchicoside + standard treatment” group and 5 patients in the “standard treatment” group reported an adverse gastrointestinal event: mild dyspepsia, nausea, and/or diarrhoea. Irritability (“nervousness”) was reported by one patient in the “thiocolchicoside + standard treatment” group and by in one patient in the “standard treatment” group. It was not determined if adverse events reported by patients in group 2 were caused by thiocolchicoside or by the standard treatment. None of the adverse events required stopping treatment [9].
Desai 2011 [20]	n/a	n/a	Not reported.
Iliopoulos 2023 [7]	-	2 (3%)	No adverse events were reported in the patients who were administered the test treatment. There were two patients (1.5% of trial patients, 3.03% of reference product) who were presented with dizziness, which was mild in severity, in the diclofenac arm. This adverse event occurred soon after administration in both patients, was considered possibly related to treatment, and spontaneously resolved quickly thereafter. There were no withdrawals from the scheduled assessment due to adverse events [18].
Ketenci 2022 [25]	10 (7%)	5 (4%)	A total of 80% of the adverse events were classified as mild in both groups. In the intervention group, two adverse events of two patients were found to be related to the study medication which were pain and hypersensitivity of the application region. One patient in the placebo group had a serious adverse event of hospitalization due to chest pain which did not lead to any complications [19].
Marcel 1990 [8]	4 (8%)	4 (8%)	Four adverse effects were noted in the placebo group (dizziness, fatigue, diarrhea, abdominal pain) and four in the thiocolchicoside group (diarrhea twice, paresthesia, somnolence) [20].
Tüzün 2003 [9]	4 (6%)	4 (6%)	There were no significant differences between the two groups in terms of adverse events. Four adverse events were reported in each group: nausea (1), heartburn (1) and diarrhea (2) in thiocolchicoside group and nausea (1), heartburn (1), hypertension (1) and dizziness (1) in the placebo group. None of the patients was withdrawn from the study due to an adverse effect [21].
Ventura 1983 [26]	n/a	n/a	No modifications in urinary and blood parameters in both groups. In the intervention group, one participant interrupted the administration of the drug at Day 5 due to gastralgia [22].

AEs = Adverse Events.
* Plain-text, as reported in the articles.

Figure S1. Sensitivity analysis excluding results derived by topical administration from the thiocolchicoside efficacy on VAS scores at 2–3 days time-point meta-analysis.

	Experimental			Control				Mean Difference	
Study or Subgroup	Mean	SD	Total	Mean	SD	Total	Weight	IV, Random, 95% CI	Year
1.2.1 Ointment									
Ketenci 2022	4.8	1.7	139	4.9	1.8	137	0.0%	−0.10 [−0.51, 0.31]	2022
Subtotal (95% CI)			0			0		Not estimable	
Heterogeneity: Not applicable									
Test for overall effect: Not applicable									
1.2.2 Oral									
Marcel 1990	4.7	1.92	49	5.22	1.84	49	23.3%	−0.52 [−1.26, 0.22]	1990
Subtotal (95% CI)			49			49	23.3%	−0.52 [−1.26, 0.22]	
Heterogeneity: Not applicable									
Test for overall effect: Z = 1.37 (P = 0.17)									
1.2.3 IM									
T0z0n 2003	4.477	1.912	71	5.567	1.697	68	31.5%	−1.09 [−1.69, −0.49]	2003
Akhter 2017	3.67	1.8	144	4.1	1.992	144	45.2%	−0.43 [−0.87, 0.01]	2017
Subtotal (95% CI)			215			212	76.7%	−0.73 [−1.37, −0.08]	
Heterogeneity: Tau^2 = 0.15; Chi^2 = 3.03, df = 1 (P = 0.08); I^2 = 67%									
Test for overall effect: Z = 2.21 (P = 0.03)									
Total (95% CI)			264			261	100.0%	−0.66 [−1.08, −0.24]	
Heterogeneity: Tau^2 = 0.05; Chi^2 = 3.14, df = 2 (P = 0.21); I^2 = 36%									
Test for overall effect: Z = 3.09 (P = 0.002)									
Test for subgroup differences: Chi^2 = 0.17, df = 1 (P = 0.68), I^2 = 0%									

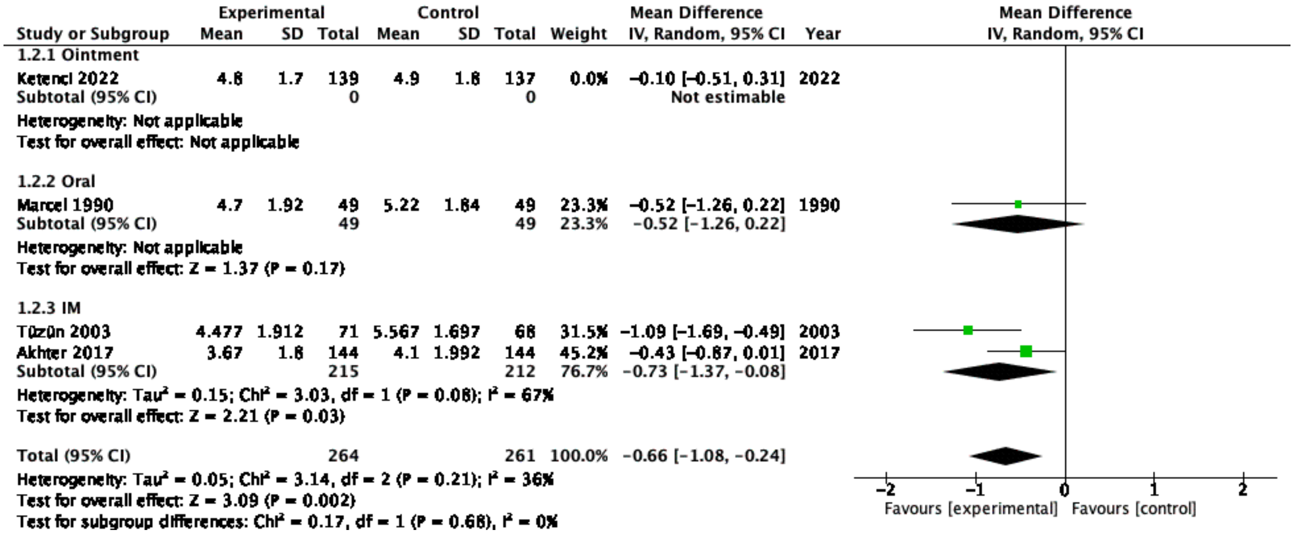

Figure S2. Sensitivity analysis excluding results derived by topical administration from the thiocolchicoside efficacy on VAS scores at 5–7 days time-point meta-analysis.

	Experimental			Control				Mean Difference	
Study or Subgroup	Mean	SD	Total	Mean	SD	Total	Weight	IV, Random, 95% CI	Year
2.2.1 Ointment									
Ketenci 2022	3.8	2.1	139	3.9	2.3	137	0.0%	−0.10 [−0.62, 0.42]	2022
Subtotal (95% CI)			0			0		Not estimable	
Heterogeneity: Not applicable									
Test for overall effect: Not applicable									
2.2.2 Oral									
Marcel 1990	2.43	2.05	49	3.63	2.25	49	19.9%	−1.20 [−2.05, −0.35]	1990
Aksoy 2002	3.3	3.7	174	4	3.8	155	20.4%	−0.70 [−1.51, 0.11]	2002
Subtotal (95% CI)			223			204	40.3%	-0.94 [-1.53, -0.35]	
Heterogeneity: Tau² = 0.00; Chi² = 0.69, df = 1 (P = 0.41); I² = 0%									
Test for overall effect: Z = 3.13 (P = 0.002)									
2.2.4 IM									
Tüzün 2003	2.505	2.092	73	4.738	1.977	68	21.8%	−2.23 [−2.90, −1.56]	2003
Desai 2011	0.985	2.53	20	1.11	2.58	20	12.8%	−0.13 [−1.71, 1.46]	2011
Akhter 2017	0.94	1.152	144	1.35	1.152	144	25.1%	−0.41 [−0.68, −0.14]	2017
Subtotal (95% CI)			237			232	59.7%	-0.99 [-2.39, 0.42]	
Heterogeneity: Tau² = 1.32; Chi² = 24.88, df = 2 (P < 0.00001); I² = 92%									
Test for overall effect: Z = 1.38 (P = 0.17)									
Total (95% CI)			460			436	100.0%	-0.99 [-1.79, -0.19]	
Heterogeneity: Tau² = 0.65; Chi² = 26.39, df = 4 (P < 0.0001); I² = 85%									
Test for overall effect: Z = 2.42 (P = 0.02)									
Test for subgroup differences: Chi² = 0.00, df = 1 (P = 0.95), I² = 0%									

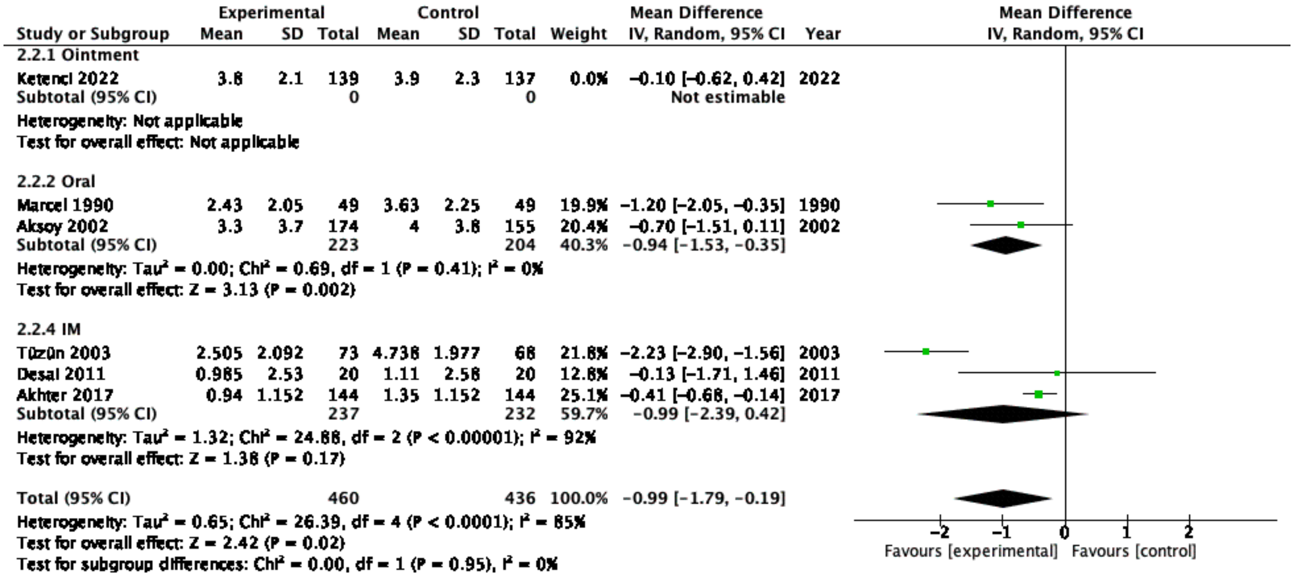

Figure S3. Sensitivity analysis excluding results derived by oral administration from the thiocolchicoside efficacy on VAS scores at 2–3 days time-point meta-analysis.

	Experimental			Control				Mean Difference	
**Study or Subgroup**	**Mean**	**SD**	**Total**	**Mean**	**SD**	**Total**	**Weight**	**IV, Random, 95% CI**	**Year**
1.2.1 Ointment									
Ketenci 2022	4.8	1.7	139	4.9	1.8	137	36.1%	−0.10 [−0.51, 0.31]	2022
Subtotal (95% CI)			139			137	36.1%	−0.10 [−0.51, 0.31]	
Heterogeneity: Not applicable									
Test for overall effect: Z = 0.47 (P = 0.64)									
1.2.2 Oral									
Marcel 1990	4.7	1.92	49	5.22	1.84	49	0.0%	−0.52 [−1.26, 0.22]	1990
Subtotal (95% CI)			0			0		Not estimable	
Heterogeneity: Not applicable									
Test for overall effect: Not applicable									
1.2.3 IM									
T0z0n 2003	4.477	1.912	71	5.567	1.697	68	28.8%	−1.09 [−1.69, −0.49]	2003
Akhter 2017	3.67	1.8	144	4.1	1.992	144	35.1%	−0.43 [−0.87, 0.01]	2017
Subtotal (95% CI)			215			212	63.9%	−0.73 [−1.37, −0.08]	
Heterogeneity: Tau² = 0.15; Chi² = 3.03, df = 1 (P = 0.08); I² = 67%									
Test for overall effect: Z = 2.21 (P = 0.03)									
Total (95% CI)			354			349	100.0%	−0.50 [−1.02, 0.02]	
Heterogeneity: Tau² = 0.15; Chi² = 7.09, df = 2 (P = 0.03); I² = 72%									
Test for overall effect: Z = 1.89 (P = 0.06)									
Test for subgroup differences: Chi² = 2.58, df = 1 (P = 0.11), I² = 61.3%									

Mean Difference
IV, Random, 95% CI

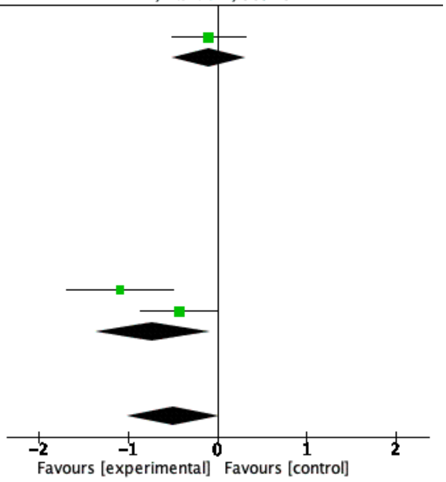

Figure S4. Sensitivity analysis excluding results derived by oral administration from the thiocolchicoside efficacy on VAS scores at 5–7 days time-point meta-analysis.

	Experimental			Control				Mean Difference	
Study or Subgroup	Mean	SD	Total	Mean	SD	Total	Weight	IV, Random, 95% CI	Year
2.2.1 Ointment									
Ketenci 2022	3.8	2.1	139	3.9	2.3	137	28.0%	−0.10 [−0.62, 0.42]	2022
Subtotal (95% CI)			139			137	28.0%	−0.10 [−0.62, 0.42]	
Heterogeneity: Not applicable									
Test for overall effect: Z = 0.38 (P = 0.71)									
2.2.2 Oral									
Marcel 1990	2.43	2.05	49	3.63	2.25	49	0.0%	−1.20 [−2.05, −0.35]	1990
Aksoy 2002	3.3	3.7	174	4	3.8	155	0.0%	−0.70 [−1.51, 0.11]	2002
Subtotal (95% CI)			0			0		Not estimable	
Heterogeneity: Not applicable									
Test for overall effect: Not applicable									
2.2.4 IM									
T0z0n 2003	2.505	2.092	73	4.738	1.977	68	26.3%	−2.23 [−2.90, −1.56]	2003
Desai 2011	0.985	2.53	20	1.11	2.58	20	15.7%	−0.13 [−1.71, 1.46]	2011
Akhter 2017	0.94	1.152	144	1.35	1.152	144	30.0%	−0.41 [−0.68, −0.14]	2017
Subtotal (95% CI)			237			232	72.0%	−0.99 [−2.39, 0.42]	
Heterogeneity: Tau² = 1.32; Chi² = 24.88, df = 2 (P < 0.00001); I² = 92%									
Test for overall effect: Z = 1.38 (P = 0.17)									
Total (95% CI)			376			369	100.0%	−0.76 [−1.65, 0.14]	
Heterogeneity: Tau² = 0.68; Chi² = 28.34, df = 3 (P < 0.00001); I² = 89%									
Test for overall effect: Z = 1.66 (P = 0.10)									
Test for subgroup differences: Chi² = 1.35, df = 1 (P = 0.25), I² = 25.9%									

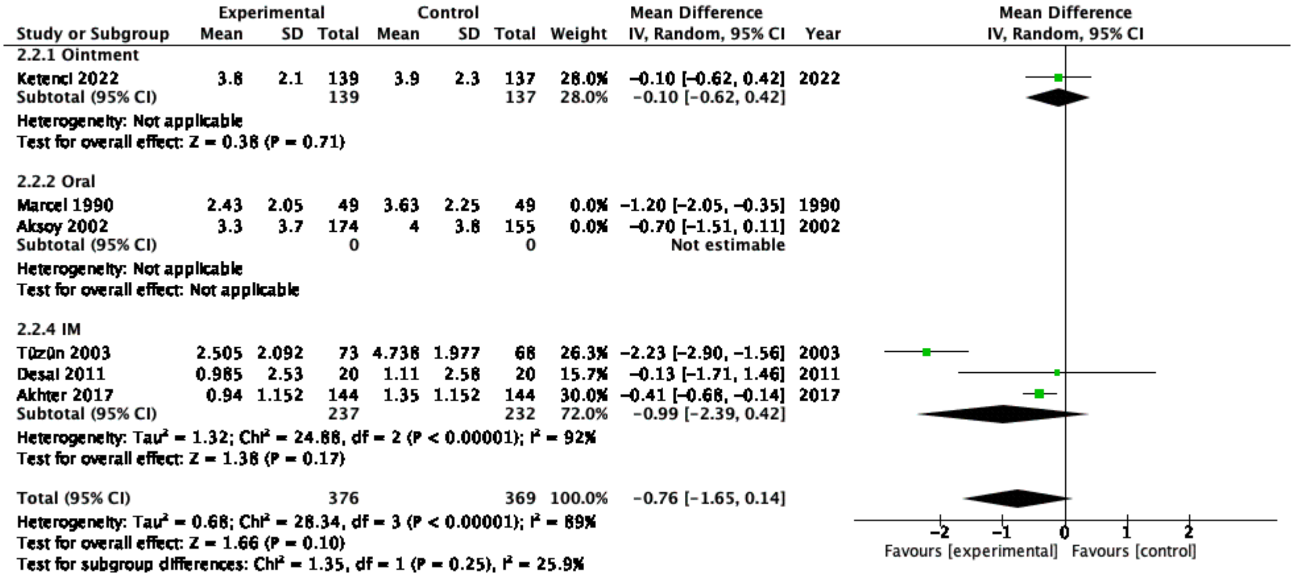

Figure S5. Sensitivity analysis excluding imputed results from Aksoy 2002 from the thiocolchicoside efficacy on VAS scores at 5–7 days time-point meta-analysis.

Study or Subgroup	Experimental Mean	Experimental SD	Experimental Total	Control Mean	Control SD	Control Total	Weight	Mean Difference IV, Random, 95% CI	Year
**2.2.1 Ointment**									
Ketenci 2022	3.8	2.1	139	3.9	2.3	137	22.7%	−0.10 [−0.62, 0.42]	2022
Subtotal (95% CI)			139			137	22.7%	−0.10 [−0.62, 0.42]	
Heterogeneity: Not applicable									
Test for overall effect: Z = 0.38 (P = 0.71)									
**2.2.2 Oral**									
Marcel 1990	2.43	2.05	49	3.63	2.25	49	19.3%	−1.20 [−2.05, −0.35]	1990
Aksoy 2002	3.3	3.7	174	4	3.8	155	0.0%	−0.70 [−1.51, 0.11]	2002
Subtotal (95% CI)			49			49	19.3%	−1.20 [−2.05, −0.35]	
Heterogeneity: Not applicable									
Test for overall effect: Z = 2.76 (P = 0.006)									
**2.2.4 IM**									
T0z0n 2003	2.505	2.092	73	4.738	1.977	68	21.2%	−2.23 [−2.90, −1.56]	2003
Desai 2011	0.985	2.53	20	1.11	2.58	20	12.1%	−0.13 [−1.71, 1.46]	2011
Akhter 2017	0.94	1.152	144	1.35	1.152	144	24.7%	−0.41 [−0.68, −0.14]	2017
Subtotal (95% CI)			237			232	58.0%	−0.99 [−2.39, 0.42]	
Heterogeneity: $Tau^2 = 1.32$; $Chi^2 = 24.88$, df = 2 ($P < 0.00001$); $I^2 = 92\%$									
Test for overall effect: Z = 1.38 (P = 0.17)									
**Total (95% CI)**			425			418	100.0%	−0.84 [−1.60, −0.09]	
Heterogeneity: $Tau^2 = 0.59$; $Chi^2 = 30.46$, df = 4 ($P < 0.00001$); $I^2 = 87\%$									
Test for overall effect: Z = 2.18 (P = 0.03)									
Test for subgroup differences: $Chi^2 = 5.28$, df = 2 (P = 0.07), $I^2 = 62.1\%$									

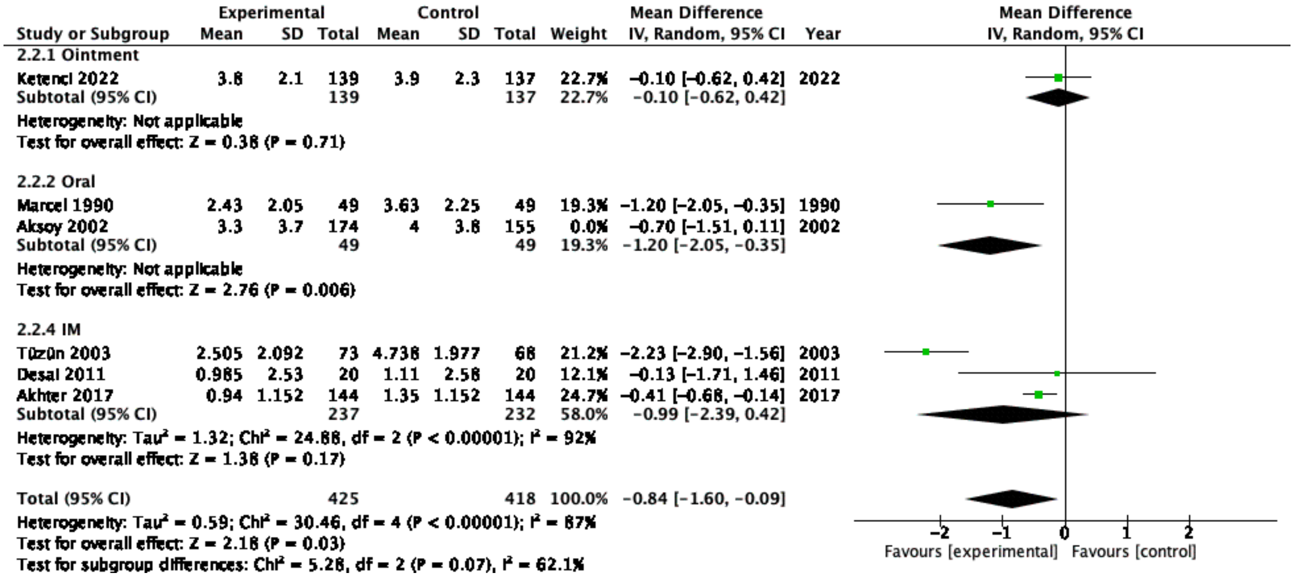

Figure S6. Sensitivity analysis excluding imputed results from Desai 2011 from the thiocolchicoside efficacy on VAS scores at 5–7 days time-point meta-analysis.

	Experimental			Control				Mean Difference	
Study or Subgroup	Mean	SD	Total	Mean	SD	Total	Weight	IV, Random, 95% CI	Year
2.2.1 Ointment									
Ketenci 2022	3.8	2.1	139	3.9	2.3	137	21.2%	−0.10 [−0.62, 0.42]	2022
Subtotal (95% CI)			139			137	21.2%	−0.10 [−0.62, 0.42]	
Heterogeneity: Not applicable									
Test for overall effect: Z = 0.38 (P = 0.71)									
2.2.2 Oral									
Marcel 1990	2.43	2.05	49	3.63	2.25	49	17.7%	−1.20 [−2.05, −0.35]	1990
Aksoy 2002	3.3	3.7	174	4	3.8	155	18.1%	−0.70 [−1.51, 0.11]	2002
Subtotal (95% CI)			223			204	35.8%	−0.94 [−1.53, −0.35]	
Heterogeneity: Tau² = 0.00; Chi² = 0.69, df = 1 (P = 0.41); I² = 0%									
Test for overall effect: Z = 3.13 (P = 0.002)									
2.2.4 IM									
T0z0n 2003	2.505	2.092	73	4.738	1.977	68	19.7%	−2.23 [−2.90, −1.56]	2003
Desai 2011	0.985	2.53	20	1.11	2.58	20	0.0%	−0.13 [−1.71, 1.46]	2011
Akhter 2017	0.94	1.152	144	1.35	1.152	144	23.3%	−0.41 [−0.68, −0.14]	2017
Subtotal (95% CI)			217			212	43.0%	−1.29 [−3.08, 0.49]	
Heterogeneity: Tau² = 1.59; Chi² = 24.46, df = 1 (P < 0.00001); I² = 96%									
Test for overall effect: Z = 1.42 (P = 0.16)									
Total (95% CI)			579			553	100.0%	−0.89 [−1.59, −0.20]	
Heterogeneity: Tau² = 0.51; Chi² = 30.18, df = 4 (P < 0.00001); I² = 87%									
Test for overall effect: Z = 2.54 (P = 0.01)									
Test for subgroup differences: Chi² = 5.17, df = 2 (P = 0.08), I² = 61.3%									

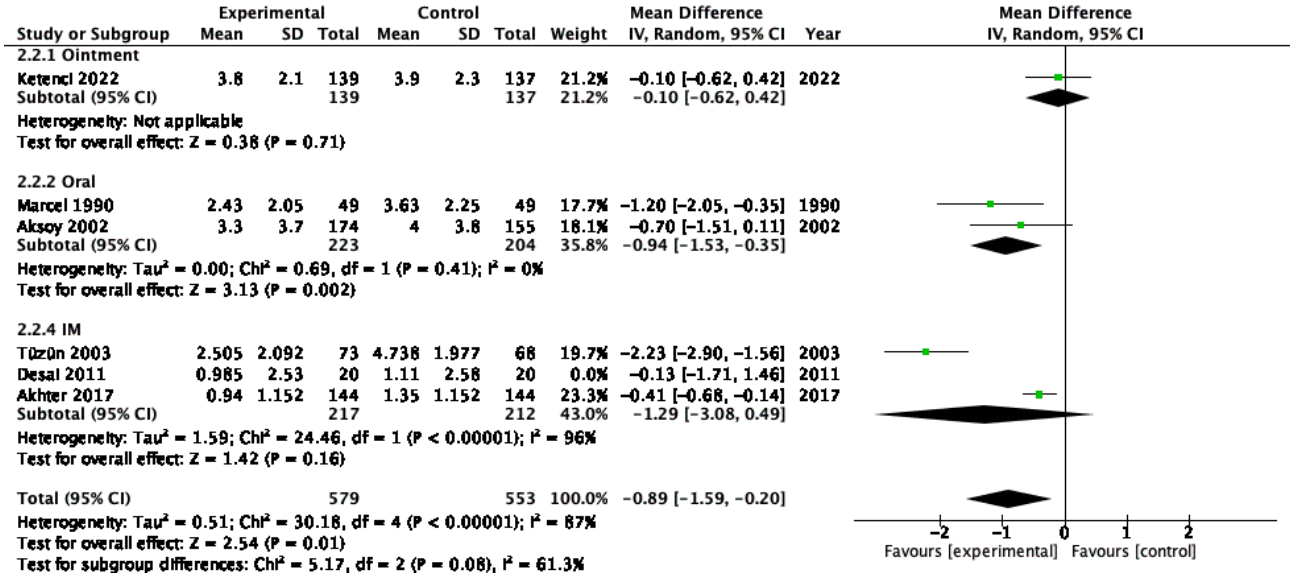

Figure S7. Sensitivity analysis excluding imputed results from Aksoy 2002 and Desai 2011 from the thiocolchicoside efficacy on VAS scores at 5–7 days time-point meta-analysis.

	Experimental			Control				Mean Difference	
Study or Subgroup	Mean	SD	Total	Mean	SD	Total	Weight	IV, Random, 95% CI	Year
2.2.1 Ointment									
Ketenci 2022	3.8	2.1	139	3.9	2.3	137	25.8%	−0.10 [−0.62, 0.42]	2022
Subtotal (95% CI)			139			137	25.8%	−0.10 [−0.62, 0.42]	
Heterogeneity: Not applicable									
Test for overall effect: Z = 0.38 (P = 0.71)									
2.2.2 Oral									
Marcel 1990	2.43	2.05	49	3.63	2.25	49	22.1%	−1.20 [−2.05, −0.35]	1990
Aksoy 2002	3.3	3.7	174	4	3.8	155	0.0%	−0.70 [−1.51, 0.11]	2002
Subtotal (95% CI)			49			49	22.1%	−1.20 [−2.05, −0.35]	
Heterogeneity: Not applicable									
Test for overall effect: Z = 2.76 (P = 0.006)									
2.2.4 IM									
Tüzün 2003	2.505	2.092	73	4.738	1.977	68	24.2%	−2.23 [−2.90, −1.56]	2003
Desai 2011	0.985	2.53	20	1.11	2.58	20	0.0%	−0.13 [−1.71, 1.46]	2011
Akhter 2017	0.94	1.152	144	1.35	1.152	144	27.9%	−0.41 [−0.68, −0.14]	2017
Subtotal (95% CI)			217			212	52.1%	−1.29 [−3.08, 0.49]	
Heterogeneity: $Tau^2 = 1.59$; $Chi^2 = 24.46$, df = 1 (P < 0.00001); $I^2 = 96\%$									
Test for overall effect: Z = 1.42 (P = 0.16)									
Total (95% CI)			405			398	100.0%	−0.95 [−1.78, −0.11]	
Heterogeneity: $Tau^2 = 0.63$; $Chi^2 = 30.12$, df = 3 (P < 0.00001); $I^2 = 90\%$									
Test for overall effect: Z = 2.22 (P = 0.03)									
Test for subgroup differences: $Chi^2 = 5.58$, df = 2 (P = 0.06), $I^2 = 64.1\%$									

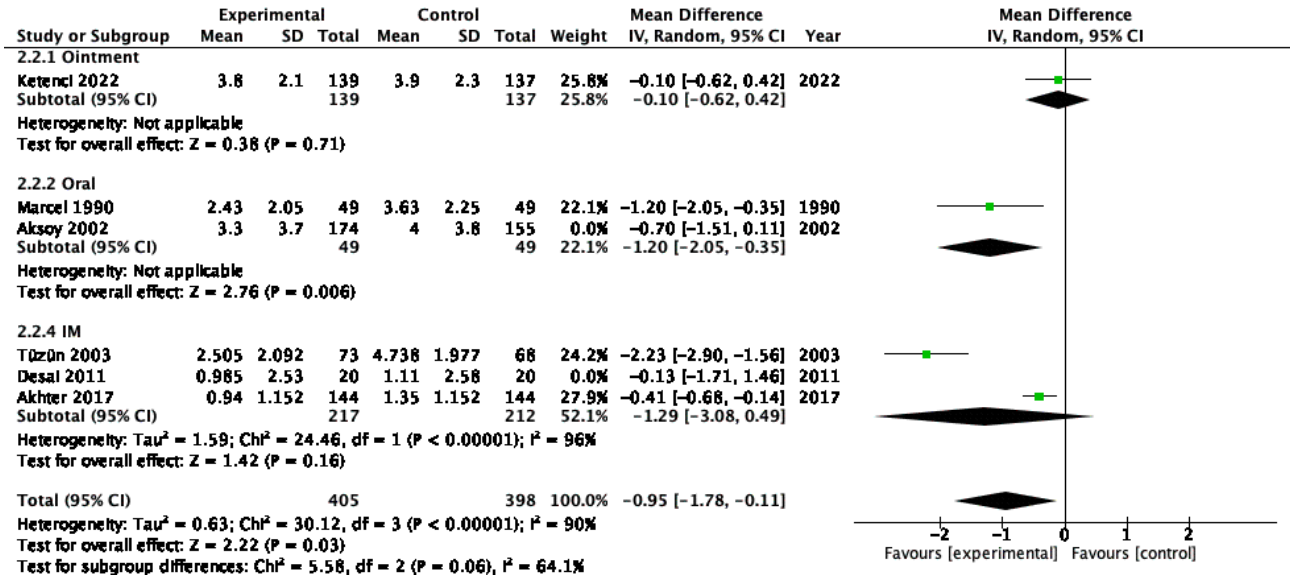

Supplement: Supplementary file 1 [file jcm-13-06133-s001.zip › jcm-3220131-supplementary.pdf]
